# Effect of louver baffles installation on hydrodynamics of bubbling fluidization in biomass gasifier

**DOI:** 10.1038/s41598-022-19120-9

**Published:** 2022-09-01

**Authors:** Kiattikhoon Phuakpunk, Benjapon Chalermsinsuwan, Suttichai Assabumrungrat

**Affiliations:** 1 Energy Research Institute, Chulalongkorn University, Phayathai Road, Wangmai, Pathumwan, Bangkok, 10330 Thailand; 2 Department of Chemical Technology, Faculty of Science, Fuels Research Center, Chulalongkorn University, Phayathai Road, Wangmai, Pathumwan, Bangkok, 10330 Thailand; 3 Center of Excellence On Petrochemical and Materials Technology, Chulalongkorn University, Phayathai Road, Wangmai, Pathumwan, Bangkok, 10330 Thailand; 4 Advanced Computational Fluid Dynamics Research Unit, Chulalongkorn University, Phayathai Road, Wangmai, Pathumwan, Bangkok, 10330 Thailand; 5 Center of Excellence On Catalysis and Catalytic Reaction Engineering, Department of Chemical Engineering, Faculty of Engineering, Chulalongkorn University, Phayathai Road, Wangmai, Pathumwan, Bangkok, 10330 Thailand; 6 Bio-Circular-Green-Economy Technology and Engineering Center, BCGeTEC, Department of Chemical Engineering, Faculty of Engineering, Chulalongkorn University, Phayathai Road, Wangmai, Pathumwan, Bangkok, 10330 Thailand

**Keywords:** Biofuels, Chemical engineering

## Abstract

Biomass gasification by a bubbling bed gasifier has been a promising process to produce fuels from biomass. However, the performance is limited by gas aggregation in the gasifier. In this study, CFD simulations were used to investigate hydrodynamics in bubbling bed gasifiers installed with multilayer louver baffles to understand the roles of baffles on different aspects including gas aggregation, biomass-bed mixing, gas-biomass contact, and pressure drop. The designed baffles could reduce the gas aggregation particularly when the biomass is fed at the middle of the baffle zone. The baffles could enhance the biomass-bed mixing and the gas-biomass contact throughout the bed except near the biomass inlet. The installation of baffles would not significantly affect the overall pressure drop but slightly affect at the mid-level of the bed. For the system in this study, the baffled gasifier with biomass feeding at the middle of the baffled zone and the gas inlet velocity of 0.7 m/s could provide the best performance in term of hydrodynamics.

## Introduction

Due to progressive increase of global energy consumption and global warming concern, biomass as a renewable source has been expected to be used as energy source for world sustainability. Gasification is a promising process for converting organic compounds such as coals and lignocellulosic biomass to synthesis gas (syngas or produced gas) consisting of mainly CO, CO_2_, H_2_ and some CH_4_. It is a thermal decomposition process in association with partial oxidation by gasifying agents like O_2_, air, steam or CO_2_^1–7^.

Gasifiers have been developed for many decades. Various designs of gasifier could be classified into 3 types^3–8^ i.e., fixed bed gasifier, fluidized bed gasifier and entrained-flow gasifier. The fixed bed gasifier which is also called as moving bed gasifier is a conventional design for biomass. Moreover, it can be divided into updraft gasifier (counter-current gasifier), downdraft gasifier (co-current gasifier) and cross-flow gasifier. The fluidized bed gasifier is also divided into bubbling bed gasifier and circulating fluidized bed gasifier. Each gasifier type has different advantages and disadvantages, as well as limitations^3,6,7,9^. Selection of suitable type and design depends on many factors such as biomass properties, operating conditions, gasifying agent, and other concerns^5–7^. However, entrained-flow gasifiers have short gas residence time because the particles and gas flow concurrently at very high velocity, and the particles must be pulverized with sizes less than 0.4 mm. Thus, the entrained-flow gasifier is likely suitable for coal rather than fibrous biomass^3–5^. At present, modern development of biomass gasifier has been focused not only on design and performance but also on challenges in development of cleaner processes^6,10,11^, applications of special techniques such as plasma gasification, supercritical water gasification and catalytic gasification^6,10,12^, large-scale integration for biorefinery^13,14^, and small-scale biomass gasification for a small community^15^.

Updraft gasifiers as well as down draft gasifiers are the oldest and simplest types. The downdraft gasifiers have less tar content in outlet gas than the updraft gasifiers. While fluidized bed gasifiers have intermediate tar content between both fixed bed types. Thus, 3/4 of gasifiers in operation have been focused on downdraft type because of their low tar production and simplicity. The running up of gasifiers has been a fluidized bed type^8^. Note that the downdraft gasifiers are inflexible to use with variety of types and particle properties of feedstocks, and unsuitable for high-moisture biomass^8,16^. Most of downdraft gasifiers have been operated with coal, which is able to control qualities of the particles, in integrated gasification combined cycle (IGCC) power plants. In case of using biomass as feedstocks, the biomass needs to be pretreated and/or pelletized before being fed in fixed bed type gasifiers^17^. Due to these problems of feedstocks, fluidized bed gasifiers become more flexible to various qualities of feed.

Bubbling bed gasifiers have many advantages such as producing uniform syngas product, getting almost uniform temperature inside, reaching the equilibrium conversion which maximizes yield with rarely unreacted carbon, and being flexible to various particle size and properties of feed like biomass. However, disadvantages of bubbling bed gasifiers include that the unreacted gas agent can pass by if bubbles are too large, and that energy input requirement might be higher than the fixed bed types because of higher velocity of gas agent^8,9,18^. Recently, the bubbling bed gasifiers using biomass as feedstock have been received more attention. In some countries, this technology has been achieved with a great leap forward via pilot plant installation^19–23^. Furthermore, there is a report indicating that bubbling bed boilers using biomass as a fuel have already been used in commercial scale in many countries such as Austria, Canada, China, Italy, Japan, and Korea^24^.

Inside the gasifier, syngas is produced from decomposition of biomass via three main steps: drying, pyrolysis and gasifying. Some of vapor (H_2_O) and volatiles (CO, CO_2_, H_2_, CH_4_ and small light hydrocarbons) can be released in the drying step and the pyrolysis step, respectively. While in the gasifying step, the syngas (CO, H_2_ and CH_4_) is formed by converting char (C) from the remaining solid with CO_2_, H_2_, H_2_O and O_2_ (mainly from inserted gasifying agent, and some from the released vapor and volatiles). In case when O_2_ or air is chosen as the gasifying agent, it also partially combusts some char, and supplies heat to the drying, pyrolysis and gasifying reactions. The O_2_ (or air) and other gasifying gases (CO_2_, H_2_O and H_2_) are then necessary to contact with the char not only to combust but also to convert C to gaseous products by Boudouard reaction (CO_2_ + C → 2CO), steam gasifying (C + H_2_O ↔ CO + H_2_) and methanation (C + 2H_2_ ↔ CH_4_)^2,9,25^. Gas–solid contact is the major key performance of the fluidized bed reactor for gas–solid reactions like fluid catalytic cracking regeneration^26^ and, especially, the gasification. However, bubbles in bed can increase their size when they flow up, and the gas–solid contacting is lower. This is caused by (1) gas formation (due to reactions) and (2) gas aggregation (due to collision of gas bubbles). In case of the gas aggregation, the higher gas inlet velocity, the bigger bubbles are formed. Thus, installation of louver baffles inside the gasifier is expected to break the gas aggregation and increase gas–solid contact by cutting size of the bubbles. In addition, the louver baffle can also reduce back-mixing of both gas and solid in some conditions^26–29^.

However, the further development of bubbling bed gasifier will need high costs via real experiments. Computational fluid dynamic (CFD) simulation is a promising technique for investigation and design development. The CFD can help to see internal phenomena that the experiments have difficulty to find inside the gasifiers. In some cases, hydrodynamic models might be conducted mutually with reliable kinetic models of gasification. Conventionally, an Euler–Euler approach with kinetic theory of granular flow (KTGF) has been a suitable standard model and widely used for simulations of fluidized bed gasification^19–23,30–32^.

The purpose of this study was to investigate effects of the designed louver baffles installed inside the bubbling bed gasifier for improving performance of gasification of biomass. CFD simulations were used to investigate hydrodynamics in the gasifier for understanding important phenomena including gas aggregation, biomass distribution, biomass-bed mixing, gas-biomass contact, and pressure drop.

## Methodology

### Mathematical models and computational methods

In this study, the Euler–Euler multiphase flow model was used for gas–solid flow simulations. In case that there was a large amount of solid particle like in fluidized bed, the kinetic theory of granular flow (KTGF) which was extended from kinetic theory of gas by adding kinetic energy oscillation owing to inelastic collisions and fluctuating motions of the particles should be applied for the solid phases^33–46^. The governing equations and constitutive models belonged to Euler–Euler model with the KTGF are shown in Tables 1 and 2.

**Table 1:** Governing equations.

**Mass conservation of phase q**	
$$\frac{\partial }{{\partial {\text{t}}}}\left( {{\upvarepsilon }_{{\text{q}}} {\uprho }_{{\text{q}}} } \right){ + }\nabla \cdot \left( {{\upvarepsilon }_{{\text{q}}} {\uprho }_{{\text{q}}} \overset{\lower0.5em\hbox{$\smash{\scriptscriptstyle\rightharpoonup}$}} {\text{v}}_{{\text{q}}} } \right){ = }\mathop \sum \limits_{{\text{p = 1}}}^{{\text{n}}} \left( {{\dot{\text{m}}}_{{{\text{pq}}}} - {\dot{\text{m}}}_{{{\text{qp}}}} } \right){\text{ + S}}_{{\text{m,q}}}$$	(1)
**Momentum conservation**	
- gas phase:	
$$\frac{\partial }{{\partial {\text{t}}}}\left( {{\upvarepsilon }_{\text{g}}{\uprho }}_{\text{g}}\overset{\lower0.5em\hbox{$\smash{\scriptscriptstyle\rightharpoonup}$}} {\text{v}}_{\text{g}}\right)+\nabla \cdot\left({{\upvarepsilon}_{\text{g}}{\uprho}}_{\text{g}}\overset{\lower0.5em\hbox{$\smash{\scriptscriptstyle\rightharpoonup}$}}{\text{v}}_{\text{g}}\overset{\lower0.5em\hbox{$\smash{\scriptscriptstyle\rightharpoonup}$}}{\text{v}}_{\text{g}}\right) = -{\upvarepsilon}_{\text{g}}\nabla{\text{p}}+\nabla\cdot\overset{\lower0.5em\hbox{$\smash{\scriptscriptstyle\rightharpoonup}$}} {\uptau}_{\text{g}}\text{+}{{\upvarepsilon}_{\text{g}}\uprho}_{\text{g}}\overset{\lower0.5em\hbox{$\smash{\scriptscriptstyle\rightharpoonup}$}} {\text{g}}\text{+}\sum_{{\text{s}}={1}}^{\text{n}}\left({\text{K}}_{\text{sg}}\left(\overset{\lower0.5em\hbox{$\smash{\scriptscriptstyle\rightharpoonup}$}} {\text{v}}_{\text{s}}-\overset{\lower0.5em\hbox{$\smash{\scriptscriptstyle\rightharpoonup}$}} {\text{v}}_{\text{g}}\right)\text{+}{\dot{\text{m}}}_{\text{sg}}\overset{\lower0.5em\hbox{$\smash{\scriptscriptstyle\rightharpoonup}$}} {\text{v}}_{\text{sg}}-{\dot{\text{m}}}_{\text{gs}}\overset{\lower0.5em\hbox{$\smash{\scriptscriptstyle\rightharpoonup}$}} {\text{v}}_{\text{gs}}\right)$$	(2)
- single solid phase:	
$$\frac{\partial }{{\partial {\text{t}}}}\left( {{\varepsilon _{\text{s}}}{\uprho _{\text{s}}}{{\overset{\lower0.5em\hbox{$\smash{\scriptscriptstyle\rightharpoonup}$}} {\text{v}} }_{\text{s}}}} \right) + \nabla \cdot \left( {{\varepsilon _{\text{s}}}{\uprho _{\text{s}}}{{\overset{\lower0.5em\hbox{$\smash{\scriptscriptstyle\rightharpoonup}$}} {\text{v}} }_{\text{s}}}{{\overset{\lower0.5em\hbox{$\smash{\scriptscriptstyle\rightharpoonup}$}} {\text{v}} }_{\text{s}}}} \right)~ = ~ - {\varepsilon _{\text{s}}}\nabla {\text{p}} - \nabla {{\text{p}}_{\text{s}}} + \nabla \cdot {\bar \uptau _{\text{s}}} + {\varepsilon _{\text{s}}}{\uprho _{\text{s}}}\overset{\lower0.5em\hbox{$\smash{\scriptscriptstyle\rightharpoonup}$}} {\text{g}} + \mathop \sum \limits_{{\text{l}} = 1}^{\text{n}} \left( {{{\text{K}}_{{\text{ls}}}}\left( {{{\overset{\lower0.5em\hbox{$\smash{\scriptscriptstyle\rightharpoonup}$}} {\text{v}} }_{\text{l}}} - {{\overset{\lower0.5em\hbox{$\smash{\scriptscriptstyle\rightharpoonup}$}} {\text{v}} }_{\text{s}}}} \right) + {{\dot {\text{m}}}_{{\text{ls}}}}{{\overset{\lower0.5em\hbox{$\smash{\scriptscriptstyle\rightharpoonup}$}} {\text{v}} }_{{\text{ls}}}} - {{\dot{\text{m}}}_{{\text{sl}}}}{{\overset{\lower0.5em\hbox{$\smash{\scriptscriptstyle\rightharpoonup}$}} {\text{v}} }_{{\text{sl}}}}} \right)$$	(3)
**Energy conservation**	
- gas phase:	
$$\frac{\partial }{{\partial {\text{t}}}}\left( {{\upvarepsilon _{\text{g}}}{\uprho _{\text{g}}}{{\text{H}}_{\text{g}}}} \right) + \nabla \cdot \left( {{\upvarepsilon _{\text{g}}}{\uprho _{\text{g}}}{{\overset{\lower0.5em\hbox{$\smash{\scriptscriptstyle\rightharpoonup}$}} {{\text{v}}} }_{\text{g}}}{{\text{H}}_{\text{g}}}} \right)~ = ~{\upvarepsilon _{\text{g}}}\frac{{\partial {{\text{p}}_{\text{g}}}}}{{\partial {\text{t}}}} + {\bar \uptau _{\text{g}}}:\nabla {\overset{\lower0.5em\hbox{$\smash{\scriptscriptstyle\rightharpoonup}$}} {{\text{v}}} _{\text{g}}} - \nabla \cdot {\overset{\lower0.5em\hbox{$\smash{\scriptscriptstyle\rightharpoonup}$}} {{\text{q}}} _{\text{g}}} + {{\text{S}}_{{\text{h}},{\text{g}}}} + \mathop \sum \limits_{{\text{s}} = 1}^{\text{n}} \left( {{{\text{Q}}_{{\text{sg}}}} + {{\dot {\text{m}}}_{{\text{sg}}}}{{\text{h}}_{{\text{sg}}}} - {{\dot {\text{m}}}_{{\text{gs}}}}{{\text{h}}_{{\text{gs}}}}} \right)$$	(4)
- single solid phase (kinetic fluctuation):	
$$\frac{3}{2}\left[ {\frac{\partial }{{\partial {\text{t}}}}\left( {{\upvarepsilon_{\text{s}}}{\uprho _{\text{s}}}{\Theta _{\text{s}}}} \right) + \nabla \cdot \left( {{\upvarepsilon_{\text{s}}}{\uprho _{\text{s}}}{{\overset{\lower0.5em\hbox{$\smash{\scriptscriptstyle\rightharpoonup}$}} {{\text{v}}} }_{\text{s}}}{\Theta _{\text{s}}}} \right)} \right]~ = ~\left( { - {{\text{p}}_{\text{s}}}\bar {\text{I}} + {{\bar \uptau }_{\text{s}}}} \right):\nabla {\overset{\lower0.5em\hbox{$\smash{\scriptscriptstyle\rightharpoonup}$}} {{\text{v}}} _{\text{s}}} + \nabla \cdot \left( {{\text{k}_{{\Theta _{\text{s}}}}}\nabla {\Theta _{\text{s}}}} \right) - {\upgamma _{{\Theta _{\text{s}}}}} + {\phi _{{\text{ls}}}}$$	(5)
$${\upgamma }_{{{\Theta }_{{\text{s}}} }} { = }\frac{{{12}\left( {{1} - {\text{e}}_{{{\text{ss}}}}^{{2}} } \right){\text{g}}_{{\text{0,ss}}} }}{{{\text{d}}_{{\text{s}}} \sqrt {\uppi } }}{\uprho }_{{\text{s}}} {\upvarepsilon }_{{\text{s}}}^{{2}} {\Theta }_{{\text{s}}}^{{{\raise0.7ex\hbox{${3}$} \!\mathord{\left/ {\vphantom {{3} {2}}}\right.\kern-\nulldelimiterspace} \!\lower0.7ex\hbox{${2}$}}}}$$ (Lun et al*.*^47^)	(6)
$$\phi_{{{\text{ls}}}} = - {\text{3K}}_{{{\text{ls}}}} {\Theta }_{{\text{s}}}$$	(7)
**Chemical species conservation of specie k in phase q**	
$$\frac{\partial }{{\partial {\text{t}}}}\left( {{\upvarepsilon ^{\text{q}}}{\uprho ^{\text{q}}}{\text{Y}}_{\text{k}}^{\text{q}}} \right) + \nabla \cdot \left( {{\upvarepsilon ^{\text{q}}}{\uprho ^{\text{q}}}{{\overset{\lower0.5em\hbox{$\smash{\scriptscriptstyle\rightharpoonup}$}} {{\text{v}}} }^{\text{q}}}{\text{Y}}_{\text{k}}^{\text{q}}} \right)~ = ~ - \nabla \cdot \left( {{\upvarepsilon ^{\text{q}}}\overset{\lower0.5em\hbox{$\smash{\scriptscriptstyle\rightharpoonup}$}} {{\text{J}}} _{\text{k}}^{\text{q}}} \right) + {\upvarepsilon ^{\text{q}}}{\text{R}}_{\text{k}}^{\text{q}} + {\upvarepsilon ^{\text{q}}}{\text{S}}_{\text{k}}^{\text{q}} + \mathop \sum \limits_{{\text{p}} = 1}^{\text{n}} \left( {{{\dot {\text{m}}}_{{{\text{p}}^{\text{k}}}{{\text{q}}^\text{k}}}} - {{\dot {\text{m}}}_{{{\text{q}}^{\text{k}}}{{\text{p}}^{\text{k}}}}}} \right)$$	(8)

Subscripts and superscripts reference:

q = any single phase, p = another single phase besides q.

g = gas phase, s = single solid phase, l = another single phase (gas or another solid phase) besides s.

k = a specie in the phase.

**Table 2:** Constitutive models.

**Stress tensor of phase q**	
$${\bar \uptau _{\text{q}}}~ = ~{\upvarepsilon _{\text{q}}}{\upmu _{\text{q}}}\left( {\nabla {{\overset{\lower0.5em\hbox{$\smash{\scriptscriptstyle\rightharpoonup}$}} {\text{v}} }_{\text{q}}} + \nabla \overset{\lower0.5em\hbox{$\smash{\scriptscriptstyle\rightharpoonup}$}}{\text{v}} _{\text{q}}^{\text{T}}} \right) + {\varepsilon _{\text{q}}}\left( {{\lambda _{\text{q}}} - \frac{2}{3}{\upmu _{\text{q}}}} \right)\nabla \cdot {\overset{\lower0.5em\hbox{$\smash{\scriptscriptstyle\rightharpoonup}$}} {\text{v}} _{\text{q}}}\bar {\text{I}}$$	(9)
**Solid shear viscosity**	
$$\upmu _{{\text{s}}} = \upmu _{{{\text{s}},{\text{col}}}} + \upmu _{{{\text{s}}.{\text{kin}}}} + \upmu _{{{\text{s}},{\text{fr}}}}$$	(10)
$${\upmu }_{{\text{s,col}}} { = }\frac{{4}}{{5}}{\upvarepsilon }_{{\text{s}}} {\uprho }_{{\text{s}}} {\text{d}}_{{\text{s}}} {\text{g}}_{{\text{0,ss}}} \left( {{1 + \text{e}}_{{{\text{ss}}}} } \right)\left( {\frac{{{\Theta }_{{\text{s}}} }}{{\uppi }}} \right)^{{{\raise0.7ex\hbox{${1}$} \!\mathord{\left/ {\vphantom {{1} {2}}}\right.\kern-\nulldelimiterspace} \!\lower0.7ex\hbox{${2}$}}}} {\upvarepsilon }_{{\text{s}}}$$	(11)
${\upmu _{{\text{s}},{\text{kin}}}}~ = ~\frac{{10{\uprho _{\text{s}}}{{\text{d}}_{\text{s}}}\sqrt {{\Theta _s}\pi } }}{{96{\upvarepsilon _{\text{s}}}\left( {{{1 + \text{e}}_{{\text{ss}}}}} \right){\text{g}_{0,{\text{ss}}}}}}{\left[ {1 + \frac{4}{5}{\upvarepsilon _{\text{s}}}{{\text{g}}_{0,{\text{ss}}}}\left( {1 + {{\text{e}}_{{\text{ss}}}}} \right)} \right]^2}{\upvarepsilon _{\text{s}}}$ (Gidaspow et al.^47^)	(12)
**Solid bulk viscosity**	
$${\uplambda }_{{\text{s}}} { = }\frac{{4}}{{3}}{\upvarepsilon }_{{\text{s}}}^{{2}} {\uprho }_{{\text{s}}} {\text{d}}_{{\text{s}}} {\text{g}}_{{\text{0,ss}}} \left( {{1 + \text{e}}_{{{\text{ss}}}} } \right)\left( {\frac{{{\Theta }_{{\text{s}}} }}{{\uppi }}} \right)^{{{\raise0.7ex\hbox{${1}$} \!\mathord{\left/ {\vphantom {{1} {2}}}\right.\kern-\nulldelimiterspace} \!\lower0.7ex\hbox{${2}$}}}}$$ (Lun et al*.*^47^)	(13)
**Solid Pressure**	
${{\text{p}}_{\text{s}}}~ = ~{\upvarepsilon _{\text{s}}}{\uprho _{\text{s}}}{\Theta _{\text{s}}} + 2\upvarepsilon _{\text{s}}^2{\uprho _{\text{s}}}{\Theta _{\text{s}}}{{\text{g}}_{0,{\text{ss}}}}\left( {1 + {{\text{e}}_{{\text{ss}}}}} \right)$ (Lun et al*.*^47^)	(14)
**Radial distribution coefficient**	
- single solid phase:	
$${\text{g}}_{{\text{0,ss}}} { = }\left[ {{1 - }\left( {\frac{{{\upvarepsilon }_{{\text{s}}} }}{{{\upvarepsilon }_{{\text{s,max}}} }}} \right)^{{{\raise0.7ex\hbox{${1}$} \!\mathord{\left/ {\vphantom {{1} {3}}}\right.\kern-\nulldelimiterspace} \!\lower0.7ex\hbox{${3}$}}}} } \right]^{{ - 1}}$$(Lun et al*.*^47^)	(15)
- mutual solid phases:	
$${\text{g}}_{{\text{0,ls}}} { = }\frac{{{\text{d}}_{{\text{s}}} {\text{g}}_{{\text{0,ll}}} + {\text{ d}}_{{\text{l}}} {\text{g}}_{{\text{0,ss}}} }}{{{\text{d}}_{{\text{s}}} +{\text{ d}}_{{\text{l}}} }}$$	(16)
**Granular temperature from KTGF**	
$$\frac{3}{2}\frac{\partial }{{\partial {\text{t}}}}\left( {{\upvarepsilon _{\text{s}}}{\uprho _{\text{s}}}{\Theta _{\text{s}}}} \right)~ = ~\left( { - {{\text{p}}_{\text{s}}}\bar {\text{I}} + {{\bar \uptau }_{\text{s}}}} \right):\nabla {\overset{\lower0.5em\hbox{$\smash{\scriptscriptstyle\rightharpoonup}$}}{\text{v}} _{\text{s}}} - {\upgamma _{{\Theta _{\text{s}}}}} + {\phi _{{\text{ls}}}}$$	(17)
**Gas–solid momentum exchange coefficient**	
- for ${\upvarepsilon }_{{\text{g}}}$ > 0.8:	
$${{\text{K}}_{{\text{sg}}}}~ = ~\frac{3}{4}{{\text{C}}_{\text{D}}}\frac{{{\upvarepsilon _{\text{s}}}{\upvarepsilon _{\text{g}}}{\uprho _{\text{g}}}\left| {{{\overset{\lower0.5em\hbox{$\smash{\scriptscriptstyle\rightharpoonup}$}} {\text{v}} }_{\text{s}}} - {{\overset{\lower0.5em\hbox{$\smash{\scriptscriptstyle\rightharpoonup}$}} {\text{v}} }_{\text{g}}}} \right|}}{{{{\text{d}}_{\text{s}}}}}{\upvarepsilon _{\text{g}}} - 2.65$$ (Gidaspow's drag model^47^)	(18)
$${\text{C}}_{{\text{D}}} { = }\frac{{{24}}}{{{\upvarepsilon }_{{\text{g}}} {\text{Re}}_{{\text{s}}} }}\left[ {{1 + 0}{\text{.15}}\left( {{\upvarepsilon }_{{\text{g}}} {\text{Re}}_{{\text{s}}} } \right)^{{{0}{\text{.687}}}} } \right]$$	(19)
- for ${\upvarepsilon }_{{\text{g}}} \le$ 0.8:	
$${{\text{K}}_{{\text{sg}}}}~ = ~150\frac{{{\upvarepsilon _{\text{s}}}\left( {1 - {\upvarepsilon _{\text{g}}}} \right){\upmu _{\text{g}}}}}{{{\upvarepsilon _{\text{g}}}d_{\text{s}}^2}} + 1.75\frac{{{\uprho _{\text{g}}}{\upvarepsilon _{\text{s}}}\left| {{{\overset{\lower0.5em\hbox{$\smash{\scriptscriptstyle\rightharpoonup}$}} {\text{v}} }_{\text{s}}} - {{\overset{\lower0.5em\hbox{$\smash{\scriptscriptstyle\rightharpoonup}$}} {\text{v}} }_{\text{g}}}} \right|}}{{{{\text{d}}_{\text{s}}}}}$$(Gidaspow's drag model^47^)	(20)
**Solid–solid momentum exchange coefficient**	
$${{\text{K}}_{{\text{ls}}}}~ \equiv ~{{\text{K}}_{{\text{sl}}}}~ = ~\frac{{3\left( {1 + {{\text{e}}_{{\text{ls}}}}} \right)\left( {\frac{\uppi }{2} + {\text{C}_{{\text{fr}},{\text{ls}}}}\frac{{{\uppi ^2}}}{8}} \right){\upvarepsilon _{\text{s}}}{\uprho _{\text{s}}}{\upvarepsilon _{\text{l}}}{\uprho _{\text{l}}}{{\left( {{{\text{d}}_{\text{l}}} + {{\text{d}}_{\text{s}}}} \right)}^2}{{\text{g}}_{0,{\text{ls}}}}}}{{2\pi \left( {{\uprho _{\text{l}}}{\text{d}}_{\text{l}}^3 + {\uprho _{\text{s}}}{\text{d}}_{\text{s}}^3} \right)}}\left| {{{\overset{\lower0.5em\hbox{$\smash{\scriptscriptstyle\rightharpoonup}$}} {{\text{v}}} }_{\text{l}}} - {{\overset{\lower0.5em\hbox{$\smash{\scriptscriptstyle\rightharpoonup}$}} {{\text{v}}} }_{\text{s}}}} \right|$$	(21)
**Gas–solid heat exchange coefficient**	
$${\text{h}}_{{{\text{sg}}}} { } \equiv {\text{ h}}_{{{\text{gs}}}} { = }\frac{{{\text{k}}_{{\text{g}}} {\text{Nu}}_{{\text{s}}} }}{{{\text{d}}_{{\text{s}}} }}$$	(22)
${\text{N}}{{\text{u}}_{\text{s}}}~ = ~\left( {7 - 10{\upvarepsilon _{\text{g}}} + 5\upvarepsilon _{\text{g}}^2} \right)\left( {1 + 0.7\operatorname{Re} _{\text{s}}^{0.2}{{\Pr }^{1/3}}} \right) + \left( {1.33 - 2.4{\upvarepsilon _{\text{g}}} + 1.2\upvarepsilon _{\text{g}}^2} \right)\operatorname{Re} _{\text{s}}^{0.7}{\text{P}}{{\text{r}}^{1/3}}$ (Gunn’s model^47^)	(23)

Herein, the CFD models would be calculated in a 2D system in transient mode. Even though the results from a 3D model are more accuracy than a 2D model, the 3D model consumes much more computational demands and time than the 2D model, especially for transient simulations. In addition, in many cases especially in cylindrical geometry like the riser, the 2D model can give sufficiently accurate results^33,48–52^.

The configurations of the system were drawn by ANSYS® DesignModeler™ and meshed by ANSYS® Meshing™. The calculations would be performed by ANSYS® Fluent®. Three phases including gas, biomass and bed phases were set, while N_2_ was selected as the inert gas in the system. The system would be operated at 800 °C and atmospheric pressure with adiabatic and no-slip wall. Hydrodynamic properties and system conditions were summarized in Table 3. All calculations were run with a time step of 1 × 10^–3^ s.

**Table 3:** Hydrodynamic properties and system conditions used in the simulations.

Properties/condition	Value/type
**Phase properties**	
Inlet granular temperature of biomass	1 × 10^5^ m^2^/s^2^
Packing limit of solid phases	0.65
Restitution coefficient of all phase interactions	0.90
**System conditions**	
Outlet pressure	1 atm
Shear condition	No slip
Inlet temperature	50 °C
Wall	Adiabatic
Operating temperature	800 °C

### Gasifier designs and conditions

**Figure 1:**
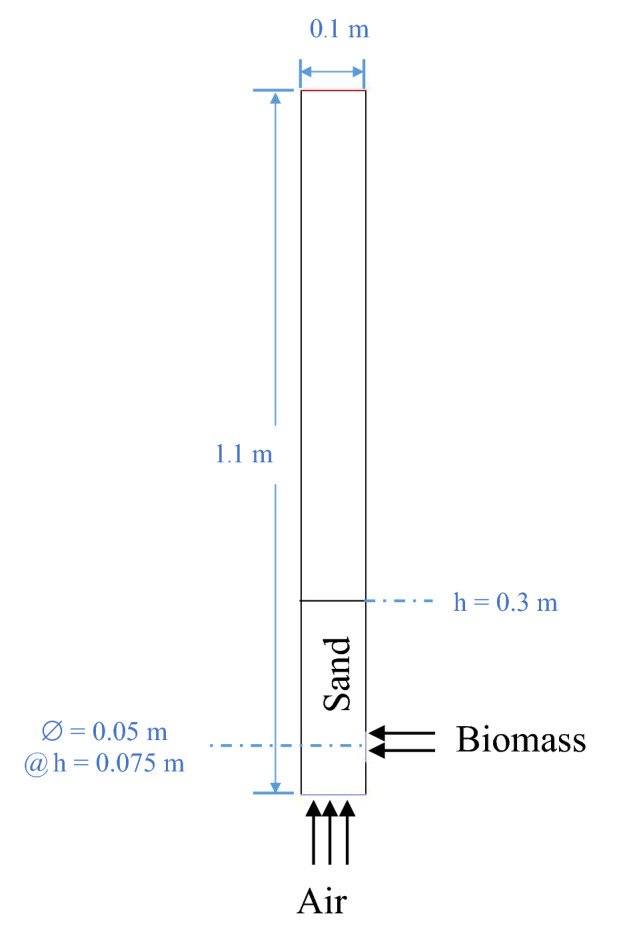
2D Schematic diagram of the bubbling bed gasifier (base design).

The base design of the bubbling bed gasifier in this study was 2D configuration with the scale of 0.1 m diameter referred to a work of Gerber et al.^30^ as shown in Fig. 1. Height of the gasifier was 1.1 m that adequate to keep freeboard zone, and height of stagnant bed was 0.3 m. Channel of biomass feed had 5 cm diameter and its center line was at 0.075 m level above the bottom (or top of gas distributor) where air was injected upwardly. Gas (air) inlet velocity would be varied between 0.5 and 0.9 m/s as shown in Table 4. To keep the same air to fuel ratio of 1.269, or equivalence ratio (ER) of 0.26, biomass inlet velocity would be varied between 0.00240 and 0.00432 m/s. The properties of solids (biomass and bed) are shown in Table 5. There was a difference between a case for validation and cases for this proposed study that the bed in validation was char, but the studied bed was sand.

**Table 4:** Gas inlet velocity and biomass inlet velocity investigated in the simulations.

Air inlet velocity	Biomass inlet velocity	Air–fuel ratio	ER
(m/s)	(m/s)	(mol/mol)	(−)
0.5	0.00240	1.269	0.26
0.7	0.00336	1.269	0.26
0.9	0.00432	1.269	0.26

**Table 5:** Physical properties of solids used in the simulations.

Properties	Value
**Biomass**	
Density of wood	585 kg/m^3^
Specific heat of wood	2380 J/ kg⋅K
Average diameter size of wood	4 mm
**Bed**	
Density of char*	450 kg/m^3^
Specific heat of char*	1600 J/ kg⋅K
Average diameter size of char*	2 mm
Density of sand	1596 kg/m^3^
Specific heat of sand	830 J/ kg⋅K
Average diameter size of sand	1 mm

*Char was active bed used only for validation with the experiment of Gerber et al.^30^.

**Figure 2:**
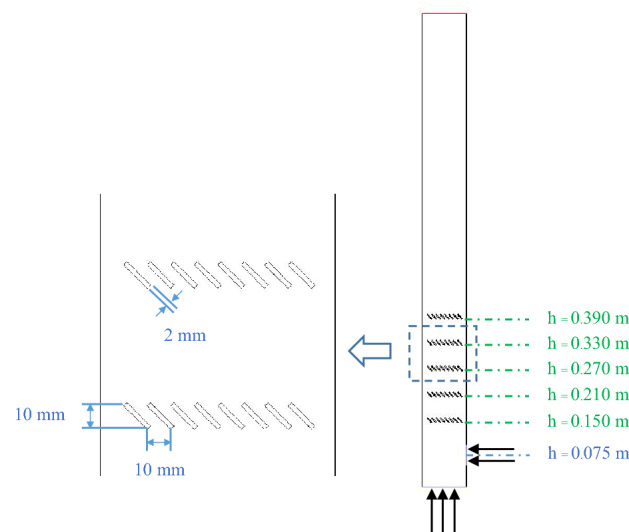
Configuration of the bubbling bed gasifier installed with louver baffles.

On the purpose of this study, the louver baffles would be installed in the bed zone above the biomass-feed level where gas formation would occur. Multilayers of the louver baffles were settled to cover the expected top of bed when operating at the highest gas inlet velocity. Herein, five layers of the louver baffles were installed at levels of 0.15 m up to 0.39 m as shown in Fig. 2. Different level between each layer was set as 0.06 m and height of each layer was 1 cm (a space between adjacent layers was 0.05 m height). Every louver blade was 45° tilted and had 2 mm thickness. Cross-sectional gap between blades was 1 cm.

### Key performance parameters

On the purposes of this study, the gas aggregation, the biomass distribution, the biomass-bed mixing, the gas-biomass contact, and the pressure drop would be investigated. The biomass distribution could be considered by volume fraction of biomass, while the pressure drops in the bed and across the bed would also be considered by pressure profile. But the gas aggregation, the biomass-bed mixing, and the gas-biomass contact could not be obviously investigated from the results from simulation data or profiles. The gas aggregation would be recognized as maximum of cross-sectional averaged voidage (volume fraction of gas) in bed zone. While the biomass-bed mixing and gas-biomass contacting would be represented by Mixing index and Contact index, respectively, defined as follows:

- Mixing index (MI) is a value of volumetric ratio of biomass to bed at the point i per the average of all points
  24 $${\text{MI}}_{{\text{i}}} { } \equiv { }\frac{{{\text{vol}}{\text{. biomass/bed at point i}}}}{{{\text{averaged vol}}{\text{. biomass/bed}}}}{ = }\frac{{{\text{n}}\left( {{\text{ VF}}_{{{\text{bm}}}} /{\text{ VF}}_{{{\text{bed}}}} } \right)_{{\text{i}}} }}{{\mathop \sum \nolimits_{{\text{i = 0}}}^{{\text{n}}} \left( {{\text{ VF}}_{{{\text{bm}}}} /{\text{ VF}}_{{{\text{bed}}}} } \right)_{{\text{i}}} }}$$
- Contact index (CI) is a value of volumetric ratio of biomass to gas at the point i per the average of all points
  25 $${\text{CI}}_{{\text{i}}} { } \equiv { }\frac{{{\text{vol}}{\text{. biomass/gas at point i}}}}{{{\text{averaged vol}}{\text{. biomass/gas}}}}{ = }\frac{{{\text{n}}\left( {{\text{ VF}}_{{{\text{bm}}}} /{\text{ VF}}_{{{\text{gas}}}} } \right)_{{\text{i}}} }}{{\mathop \sum \nolimits_{{\text{i = 0}}}^{{\text{n}}} \left( {{\text{ VF}}_{{{\text{bm}}}} /{\text{ VF}}_{{{\text{gas}}}} } \right)_{{\text{i}}} }}$$

The MI indicates the distribution of biomass mixed in the bed, while the CI indicates the distribution of gas-biomass contacting. If a value/profile of MI or CI is close to 1 that means the biomass-bed mixing or the gas-biomass contacting is well distributed.

Both MI and CI at local points in the bed zone would be plotted and created contours to be investigated. In addition, averages of MI and CI in x- or y-direction would be plotted to investigate their changes in vertical and horizontal directions.

## Grid refinement and validation

**Figure 3:**
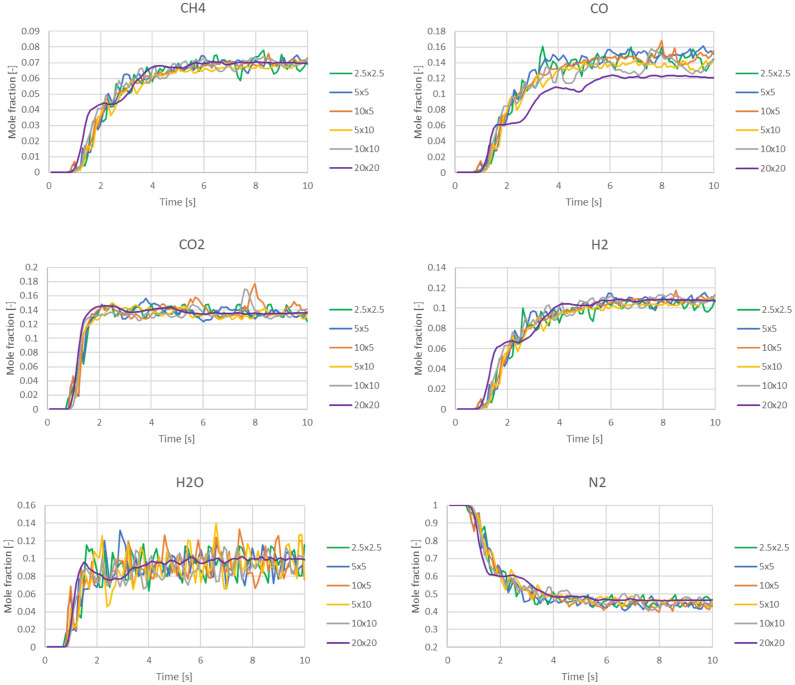
Time-dependent mole fractions of product gases at different mesh sizes (dx and dy in mm) for validation with experimental data of Gerber et al.^30^.

In the previous works^53,54^ which performed 2D simulations of the fluidized bed reactor for steam reforming reaction, the models of Euler–Euler multiphase flow with the kinetic theory of granular flow (KTGF) shown in Tables 1 and 2 had been proven effective for studying fluidized bed behavior (cold flow test). It provided reliable reaction products (hot flow test) when running with kinetics. In this study, the main components of gas product were validated with experimental results of Gerber et al.^30^ who performed the gasification in a bubbling bed reactor. If the results showed good agreement, it could infer that the hydrodynamic behavior was also proven. The simulated mole fractions of product gases as a function of time were shown in Fig. 3. The grid refinement was also performed with different sizes of square mesh with dx and dy in a range of 2.5 to 20 mm. Obviously, the 20 × 20 sq mm mesh was insufficient because the fraction of all gases rarely fluctuated, differently from the other smaller meshes. Additionally, the 20 × 20 sq mm mesh showed the fraction of CO evidently lower than the others. While the other mesh sizes resulted in lines with close trend and fluctuation. This was also confirmed by the previous works^53,54^ whose preferred mesh sizes of dx and dy were not over 5 and 10 mm, respectively. Thus, both dx and dy were preferred as 5 or 10 mm for further simulations depending on significance of change in that direction. While the 2.5 × 2.5 sq mm mesh would not be appropriate due to redundant calculation time.

Next, each the mole fraction of gas component from the results with 5 × 10 sq mm mesh was averaged by 6–10 s and compared to the experimental results as shown in Table 6. The mole fractions of all gases were close to the experiments, although the mole fractions of CH_4_ and H_2_ were slightly over. Therefore, the validation could be acceptable.

**Table 6:** Time-averaged mole fraction of product gas validated with the experimental data of Gerber et al.^30^.

	Mole fraction (dry basis)				
	CH_4_	CO	CO_2_	H_2_	N_2_
**Experiments**					
Max	0.07	0.21	0.18	0.11	0.60
Min	0.02	0.12	0.13	0.08	0.48
Mean	0.05	0.18	0.15	0.10	0.52
**This simulation**	0.075	0.152	0.147	0.115	0.511

**Figure 4:**
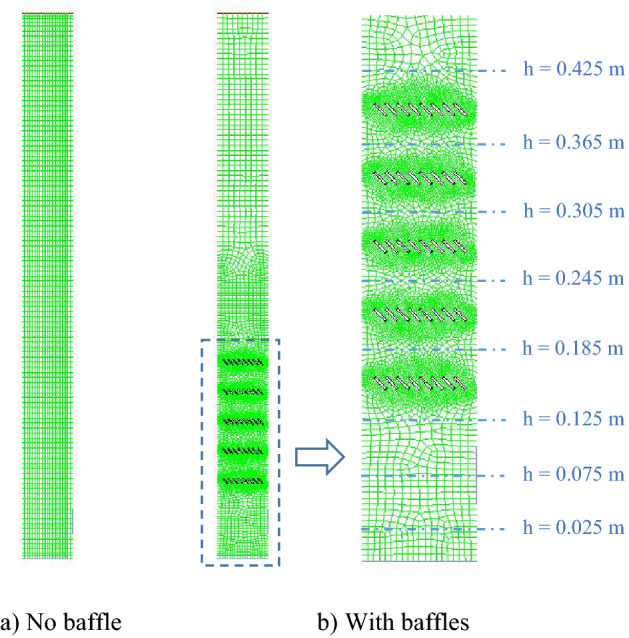
Mesh configurations of the gasifiers with and without the baffles.

For the proposed study, the 5 × 10 sq mm mesh was selected same as the previous works^53,54^ as shown in Fig. 4a, and that made the number of cells equaled to 2220. In case of the baffled gasifier in Fig. 4b, the area was divided into two zones, i.e., bed zone at bottom which covered all baffle layers and freeboard zone at top. The phenomena affected by baffles might be more significant, and therefore, the dx and dy in bed zone were selected as 5 mm and the grid on baffles’ edges was finer. Whereas the bigger mesh of 10 × 10 sq mm was applied in freeboard zone in order to reduce calculation time. The total number of cells of the baffled gasifier was reasonable at 8088. Additionally, to compare results between before and after installing the baffles, the values at the heights near the baffle layers would be averaged by cross sectional area.

## Hydrodynamic investigation

**Figure 5:**
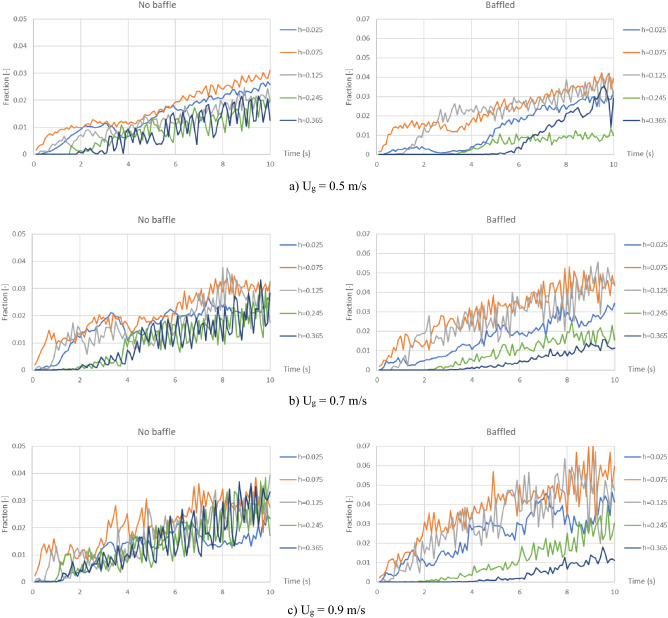
Area-averaged biomass fraction as a function of time at different heights inside the no-baffle gasifier (left) and the baffled gasifier (right), when operating at various gas inlet velocities.

At the beginning, because the simulations in this study were transient, biomass fraction would increase unlimitedly because there was no biomass conversion involved in this study and no solid out of the gasifiers. Figure 5 shows area-averaged biomass fraction at different heights covering the bed zone in all cases of different gas inlet velocities for the gasifiers before and after installing the baffles. In all graphs, almost lines of biomass fraction seemed increasing stably after 5 or 6 s. Thus, all transient results would be averaged by time in a range of 6–10 s to compare the results of each case with the others.

### Effect of baffles

**Figure 6:**
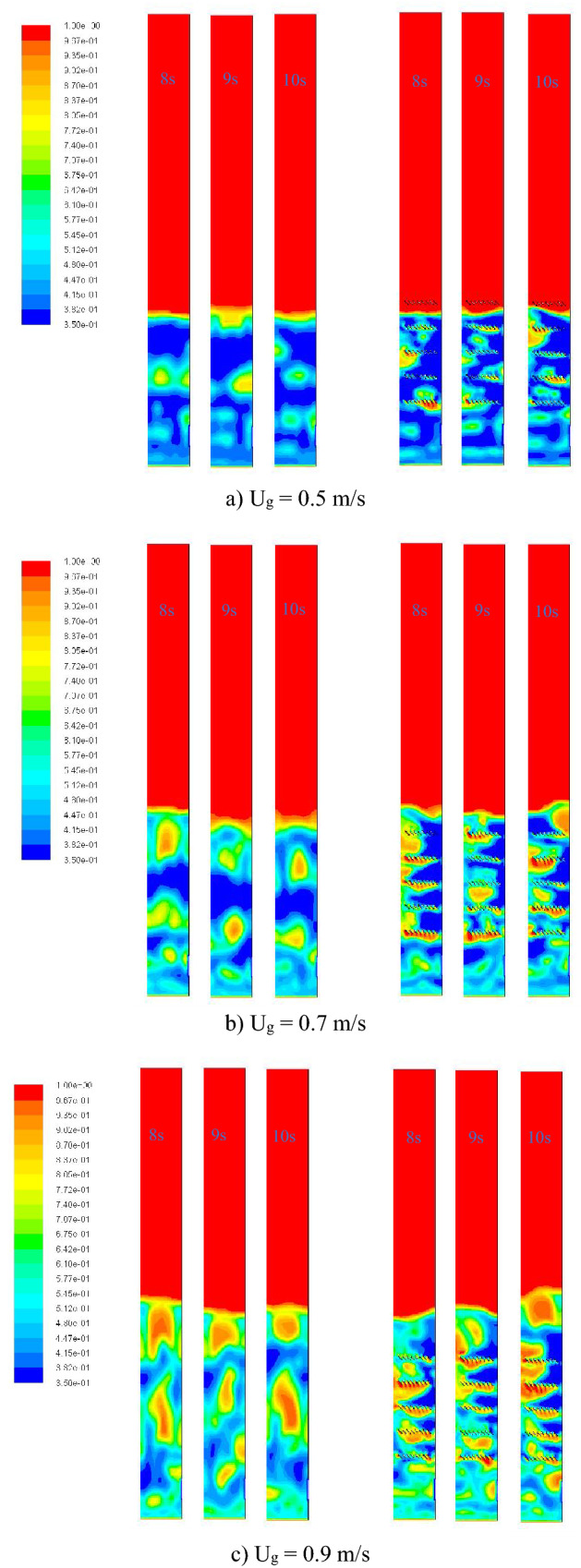
Voidage (gas fraction) contours inside the no-baffle gasifier (left) and the baffled gasifier (right) snapped at 8, 9, 10 s when operating at various gas inlet velocities.

Firstly, CFD could give the visual graphics of bubble appearance in the bed which was represented by gas fraction contours as shown in Fig. 6. The gas aggregation, which made bigger bubbles, obviously occurred in the gasifier without the baffles particularly in the cases of higher gas inlet velocities (0.7 and 0.9 m/s). On the other hand, the bubbles appeared in small size when the baffle layers were installed. Although each baffle layer could reduce size of the bubbles when they passed through, some bubbles would be obstructed and aggregated under the layer. In addition, the bed height was not changed by installing the baffles with any gas inlet velocity.

**Figure 7:**
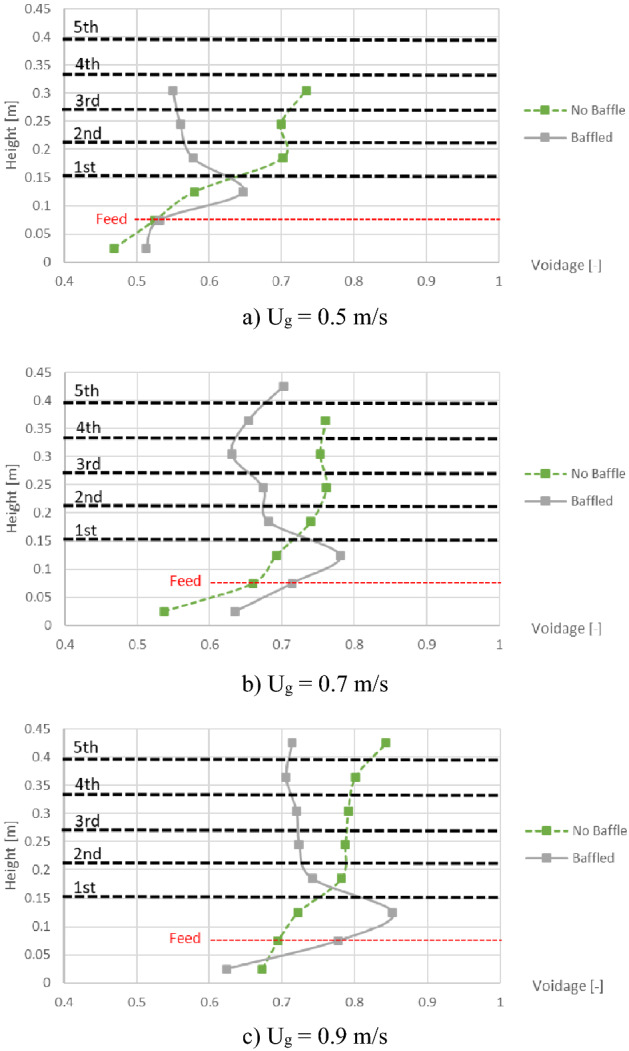
Maximum of cross-sectional averaged voidage in bed before and after installing the baffles, when operating at various gas inlet velocities.

For clarity, the maximum values of cross-sectional averaged voidage in the bed at different heights between 8 and 10 s were plotted for comparison between with and without the baffles as shown in Fig. 7. Results with all gas inlet velocities similarly indicated that the baffles could reduce the maximum voidage in any level in the baffle zone, but the maximum voidage would increase under the baffle zone. Thus, the zone in the designed baffles could be promised for better performance of the gasification.

**Figure 8:**
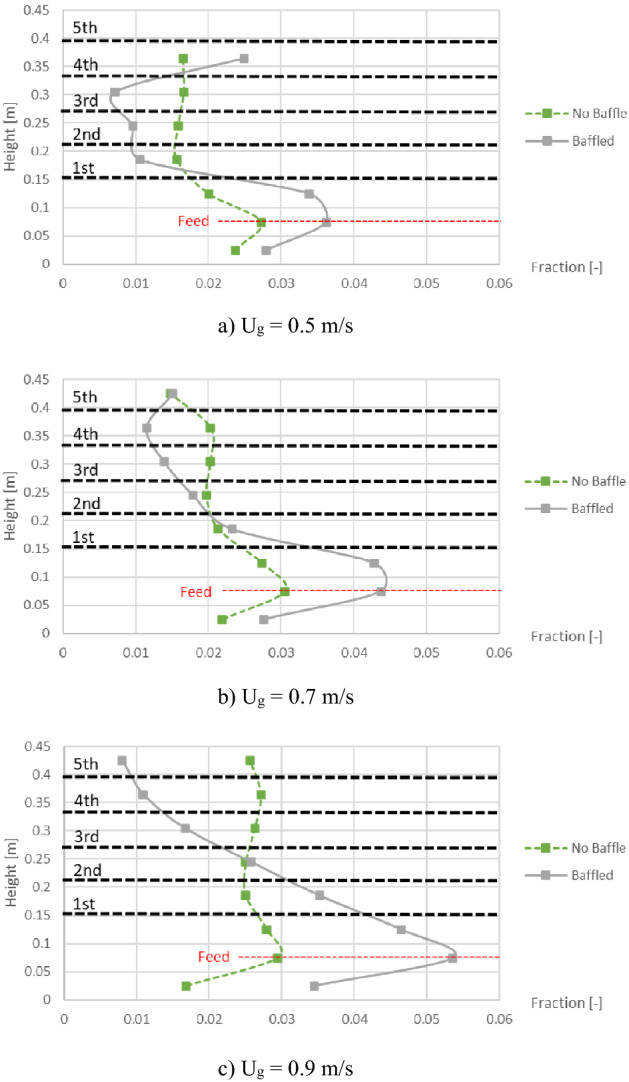
Time-and-area-averaged biomass fraction in bed before and after installing the baffles, when operating at various gas inlet velocities.

However, the distribution of biomass was another key parameter to investigate besides the gas aggregation. Profiles of area-averaged biomass fraction in bed at different heights were plotted as shown in Fig. 8. However, the results of the biomass distribution were contrast to the maximum voidage. In the baffle zone, the values of biomass fraction would decrease after installing the baffles at most of heights. While under the baffle zone where biomass was fed in, the values of biomass fraction would exactly increase after installing the baffles. This was because most biomass as well as the bubbles would be obstructed under the first baffle layer. Some biomass particles would hit the blades and hardly be lifted passing through the thin layer of gas forming under the first baffle layer as can be seen in Fig. 6.

### Modification of feed position

To prevent obstruction of biomass under the baffle zone belonging to the base design, the position of biomass feed channel was changed from the level of 0.075 m, as can be seen in Fig. 2, to a level of 0.245 m which was at the middle between the second layer and the third layer of baffles.

**Figure 9:**
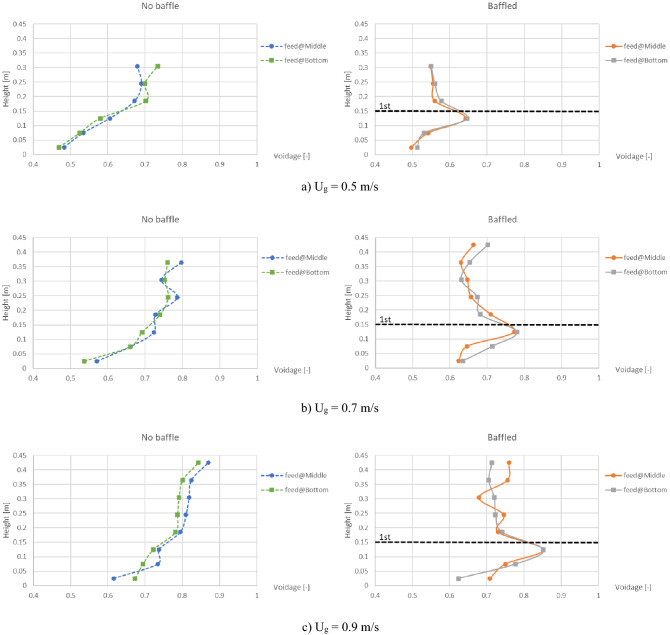
Effect of biomass feed position on maximum of cross-sectional averaged voidage in bed inside the no-baffle gasifier (left) and the baffled gasifier (right), when operating at various gas inlet velocities.

**Figure 10:**
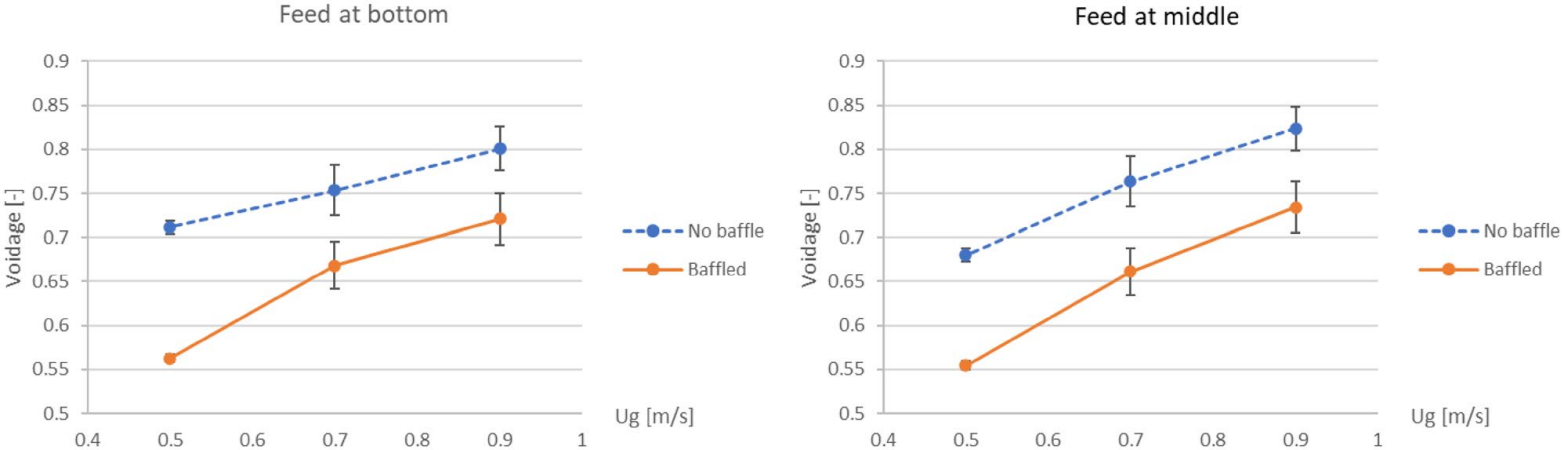
Effect of the baffles installation on maximum of cross-sectional averaged voidage in bed as a function of gas inlet velocity in cases that biomass was fed at bottom (left) and at middle (right).

The first parameter to consider was the maximum of cross-sectional averaged voidage in comparison between feeding biomass at the bottom and at the middle as shown in Fig. 9. In every gas inlet velocity, and both with and without the baffles, all profiles similarly show no significant difference between the positions of biomass feed. In addition, the maximum values of voidage were plotted as a function of gas inlet velocity as shown in Fig. 10. According to the results in Fig. 9, profiles in cases of both biomass feed positions were similar to each other and indicated that installing the baffles could reduce the maximum voidage in every gas inlet velocity. Moreover, increase of gas inlet velocity would proportionally increase the maximum voidage. Overall results from Figs. 9 and 10 indicated that the baffles could break the gas aggregation in the baffle zone whichever position of biomass feed was added.

**Figure 11:**
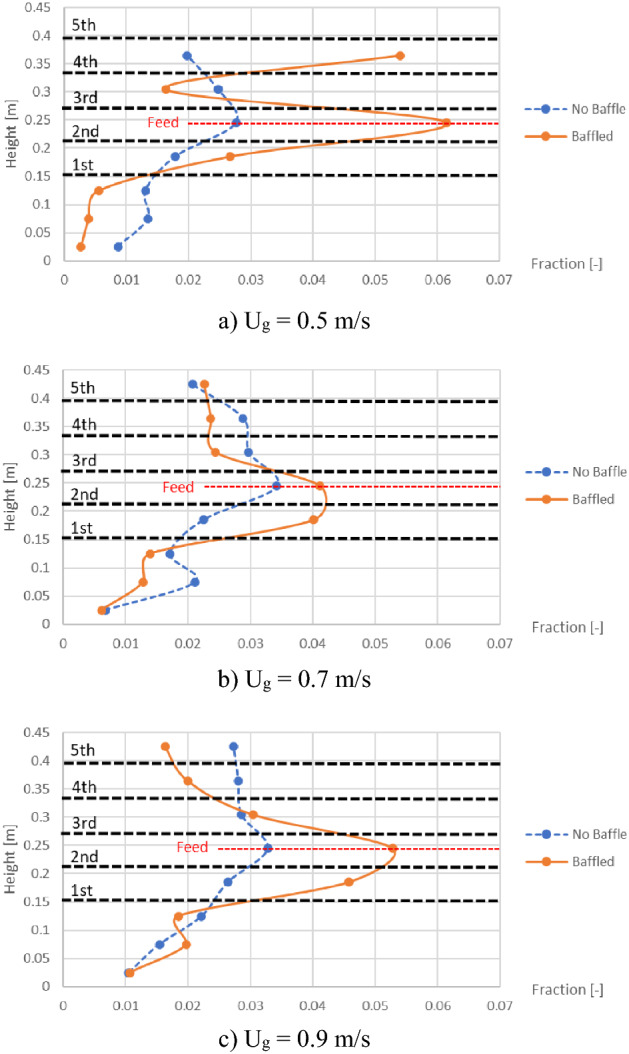
Time-and-area-averaged biomass fraction in bed before and after installing the baffles in case that biomass was fed at middle, when operating at various gas inlet velocities.

**Figure 12:**
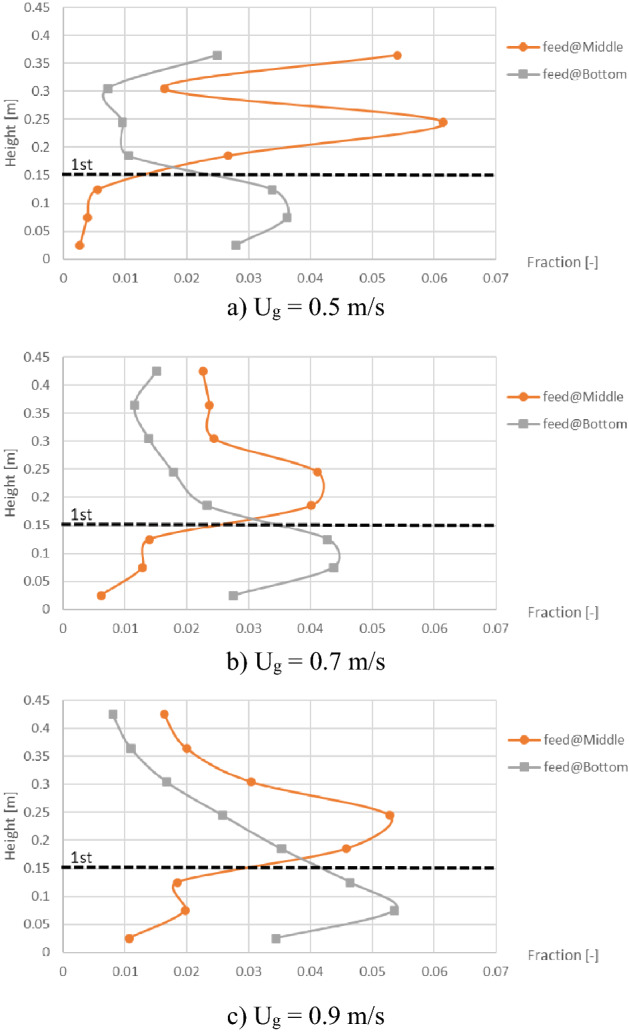
Effect of biomass feed position on time-and-area-averaged biomass fraction in case of the baffled gasifier when operating at various gas inlet velocities.

On the purpose of changing biomass feed position to the middle of the baffle zone, the biomass distribution profiles were plotted as shown in Fig. 11 compared to those in Fig. 8. In contrast to the biomass feeding (at bottom) below the baffle zone (Fig. 8), the baffles could provide higher biomass fraction at levels in the baffle zone (especially at the level of feed in). Considering only the case that the baffles were installed, comparison between both biomass feed positions on the biomass distribution was investigated as shown in Fig. 12. Comparison results with all gas inlet velocities showed that the biomass feeding at the middle position provided higher biomass fraction in the baffle zone, whereases it provided less biomass fraction in the zone below the baffles.

It could be concluded that the multilayers of baffles were capable to perform the gasification better because the gas aggregation would be reduced in the baffle zone (above the first layer of baffles). In addition, the biomass should be fed at the middle of the baffle zone to have the biomass dense in this zone.

**Figure 13:**
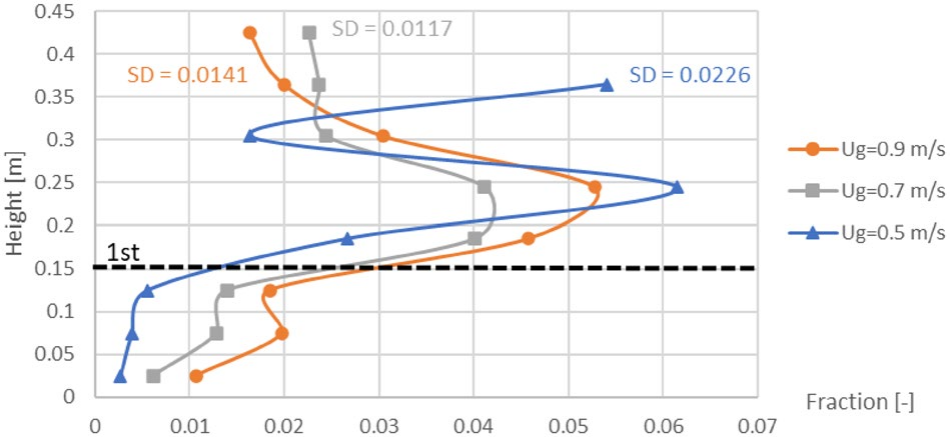
Effect of gas inlet velocity on time-and-area-averaged biomass fraction in case of the baffled gasifier with biomass feeding at middle.

Lastly, when the baffles and the biomass feeding at the middle were settled according to the purpose, the gas inlet velocities would be compared in term of biomass distribution as results in Fig. 13. Each vertical profile of averaged biomass fraction was very fluctuated per different levels in the baffles zone. Among these gas inlet velocities, the velocity of 0.7 m/s obtained the smallest variation (SD = 0.0117) and was followed closely by the velocity of 0.9 m/s (SD = 0.0141). But the velocity of 0.5 m/s obtained evidently higher variation (SD = 0.0226) which indicated that this gas velocity was insufficient to blow the biomass passed fluently through the baffle layer. However, only in the case with the velocity of 0.5 m/s, the biomass significantly bulked at the level of 0.365 m which exactly was the top of the bed. The bulked biomass phenomena would not occur when there was no baffle (seen in Fig. 11a), but it was a result of the baffles wherever the feed position was positioned (seen in Fig. 12a). In general, when the bubbles flow to top of the bed and break out, some particles will be flown in freeboard then fall. In this solid mixture, heavier solid (sand) might fall to bed faster than lighter solid (biomass). However, if next flown-up bubbles were large enough or dispersed properly, the two solids on top of bed would be mixed again. In Fig. 6a, the bubbles passing through the baffles with low gas inlet velocity of 0.5 m/s were unevenly dispersed and small, whereas big bubbles could still appear on the top with higher gas inlet velocity as seen in Figs. 6b and c. It could be inferred that insufficient gas inlet velocity did not only make the solids blew harder through the baffle layer above the feed level but also made the biomass bulked on top of the bed.

## Mixing index and contact index

**Figure 14:**
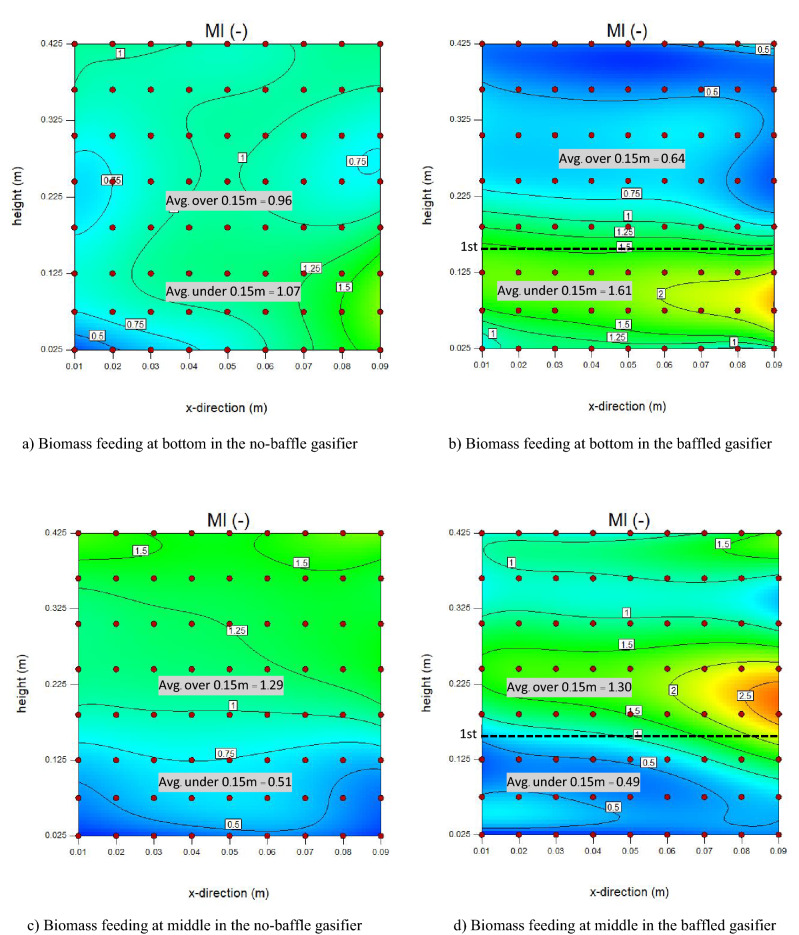
Contours of time-averaged local mixing index (MI) in case of U_g_ = 0.7 m/s.

**Figure 15:**
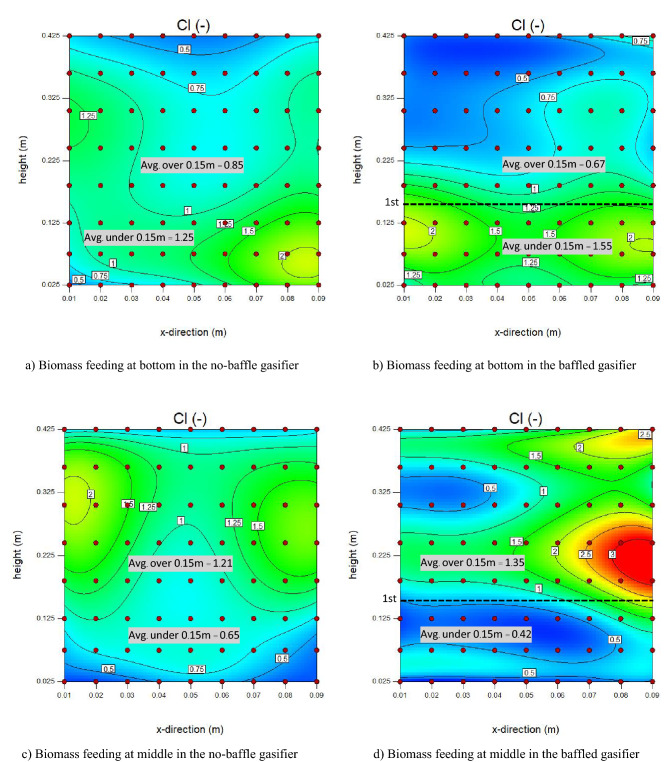
Contours of time-averaged local contact index (CI) in case of U_g_ = 0.7 m/s.

MI and CI values at 9 × 8 local points in the bed zone were calculated via Eqs. 24 and 25, respectively, and were plotted in two directions. Figures 14 and 15 show plots and contours of the local MI and CI, respectively. Herein, a gas inlet velocity of 0.7 m/s was selected as an example case.

In Fig. 14a and b representing the cases with biomass feeding at bottom, installing the baffles would make distribution of MI evidently differ to the case without installing the baffles. With the decreasing of averaged MI in the promising baffle zone from 0.96 to 0.64, while the averaged MI under the baffle zone increased from 1.07 to 1.61. This can be explained by the previous discussion in Fig. 8 that the biomass being fed below the baffle zone would be obstructed by the first baffle layer. When the biomass feed position was changed to the middle of baffle zone as shown in Fig. 14c and d, the averages of MI both in and under the baffle zone were rarely different between before and after installing the baffles. However, whichever position the biomass was fed, installing the baffles would make biomass clustered and make MI quite a bit higher near the biomass inlet.

Like the MI discussed above, averaged CI in the promising baffle zone decreased but the averaged CI under the baffle zone increased when biomass was fed at bottom as shown in Fig. 15a and b. In the same way, the averages of CI both in and under the baffle zone slightly changed after installing the baffles when biomass was fed at middle as shown in Fig. 15c and d. But the CI near the biomass inlet was over 3 when biomass was fed at middle. It meant that gasifier agent might have less contact with biomass in this spot. Moreover, the results indicated that biomass feeding at middle of the baffle zone gave advantage over biomass feeding below the baffle zone by providing averaged MI and CI in the promising baffle zone more than 1. Thus, when biomass was fed at middle in the baffled gasifier, two advantages were provided simultaneously in the same zone. One was the increasing of amount of biomass, and the other was the decreasing of unreacted gas in the core of bubbles by bubble size reducing. Both the advantages led that biomass particle to get higher probability to interact with gasifying gas. In the same way, installing the baffles together with feeding biomass into the middle of the baffle zone could provide better biomass conversion.

For clarity, the MI and CI in cases of all gas inlet velocities would be further investigated in vertical and horizontal directions in next parts.

### Vertical indices

**Figure 16:**
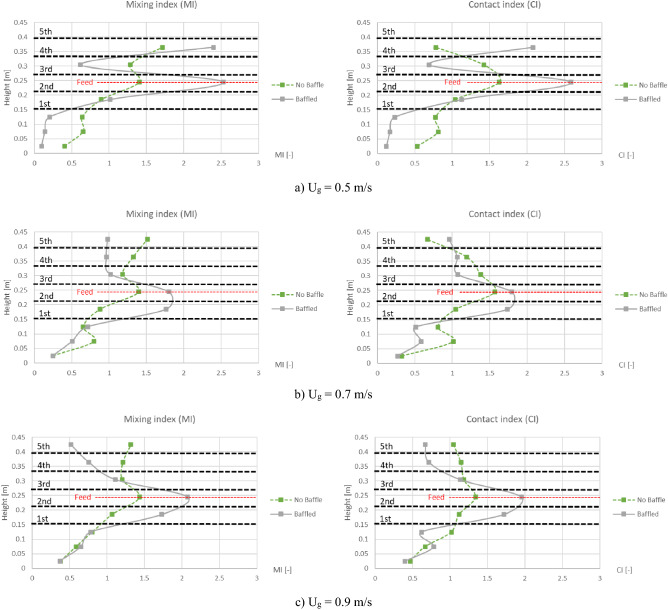
Time-and-area-averaged vertical mixing index (left) and contact index (right) before and after installing the baffles in case that biomass was fed at middle, when operating at various gas inlet velocities.

Vertical profiles of area-averaged MI and CI in comparison between with and without the baffles were plotted when the biomass was fed at middle as shown in Fig. 16. All the vertical profiles of MI were very similar to those of CI as well as the biomass distribution profiles in Fig. 11. Like previous discussion about the biomass distribution profiles, it could also explain that when the biomass was fed at middle of the baffle zone, installing the baffles could provide higher averaged MI and CI at many levels in the baffle zone (very high at the level of feed in).

**Figure 17:**
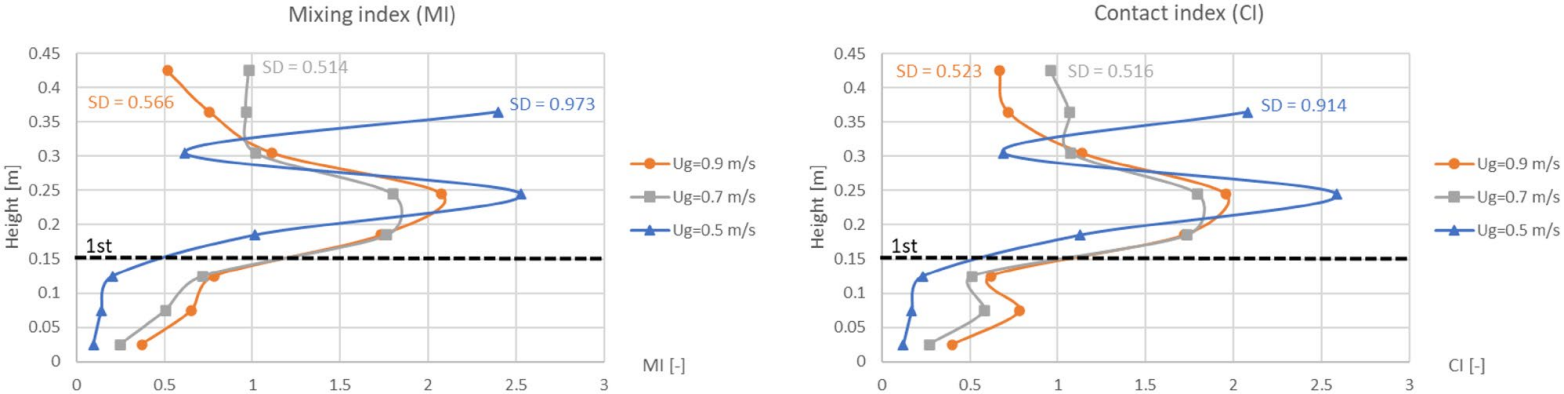
Effect of gas inlet velocity on time-and-area-averaged vertical mixing index (left) and contact index (right) in case of the baffled gasifier with biomass feeding at middle.

Even if the vertical profiles of averaged MI and CI did not indicate another point rather than those of biomass distribution, the values of the area-averaged MI and CI could describe how well they distributed (the values of MI and CI should not be much deviated from 1). When the biomass was fed at the middle, the area-averaged values of the MI and CI in Fig. 16 in all cases of gas inlet velocity evidently showed that whichever the baffles installed or not, both the averaged MI and CI were lower than 1 near the bottom or under the baffle zone but over than 1 in above zone. But with the baffles, the ranges of averaged MI and CI were wider than those without the baffles. The widest range around 0.1–2.6 belonged to the gas inlet velocity of 0.5 m/s, while the velocity of 0.9 m/s and 0.7 m/s provided the range around 0.4–2.1 and 0.3–1.8, respectively. For clarity, Fig. 17 also shows comparison of gas inlet velocities on the vertical MI and CI when the baffles were installed, and the biomass was fed at the middle. Among these gas inlet velocities, the velocity of 0.5 m/s provided the highest variation (SD = 0.973), while the velocity of 0.9 m/s and 0.7 m/s gave moderate variation (SD = 0.566 and 0.514, respectively). According to the previous results of Fig. 13, the velocity of 0.5 m/s was still unsatisfied to blow the biomass through the baffle layer. Although, the velocity of 0.7 m/s got a little less variation than the velocity of 0.9 m/s, this velocity had much less gas aggregation according to Figs. 6 and 10. Thus, it could infer that the best performance of the provided system in this study could occur with gas inlet velocity of 0.7 m/s when the designed baffles were installed with feeding biomass at the middle of baffle zone.

For other applications with different biomass or geometry/scale, the best gas inlet velocity might be deviated from 0.7 m/s because bubble formation in the bed is dependent on properties of solid and gas, and the bubble size reduction is strongly dependent on the geometry of the baffles. Therefore, when the geometry is changed, new simulations must be performed to investigate the best gas velocity case by case. But in case that the biomass and/or other properties and conditions excluding the baffle geometry are changed, the scaling law derived by Glicksman et al.^55,56^ can be applied to estimate the best gas velocity by the following dimensionless terms.

26 $$\frac{{{\text{U}}_{{\text{g}}}^{{2}} }}{{{\text{gH}}}}{, }\frac{{{\text{U}}_{{\text{g}}}^{{2}} }}{{{\text{gd}}_{{\text{s}}} }}{, }\frac{{{\text{G}}_{{\text{s}}} }}{{\rho_{{\text{s}}} {\text{U}}_{{\text{g}}} }}{, }\frac{{\rho_{{\text{g}}} {\text{U}}_{{\text{g}}} {\text{H}}}}{{\mu_{{\text{g}}} }}{, }\frac{{\rho_{{\text{g}}} {\text{U}}_{{\text{g}}} {\text{d}}_{{\text{s}}} }}{{\mu_{{\text{g}}} }}$$

However, new simulations with the different biomass are recommended to confirm the results from the scaling law. For the biomass that has density and particle size close to those of the biomass in this study, it is likely to get the best gas velocity around 0.7 m/s if they are performed in this provided system.

### Horizontal indices

**Figure 18:**
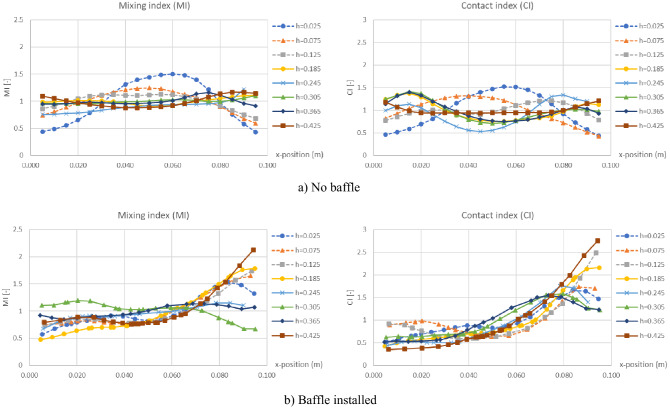
Time-averaged horizontal mixing index (left) and contact index (right) at different heights in case of biomass feeding at middle and U_g_ = 0.7 m/s.

Figure 18 shows horizontal profiles of MI and CI at different levels for both before and after installing the baffles, with a gas inlet velocity of 0.7 m/s as an example case. In case of no baffle, the horizontal profiles of both MI and CI obtained good distribution with variation of only ± 0.5. But when the baffles were installed, some MI and CI points on the right of profiles would increase over 1.5 at many levels. The increases of MI and CI near the biomass inlet were also accorded to the contours of MI and CI previously discussed in Figs. 14 and 15. However, most points of MI and CI were still in 1 ± 0.5, although the averages of MI and CI in some levels as seen in Fig. 16 might be much over 1.5 when the baffles were installed. This meant that even the baffles were installed, the MI and CI still had good distribution in most zones.

## Pressure drop

**Figure 19:**
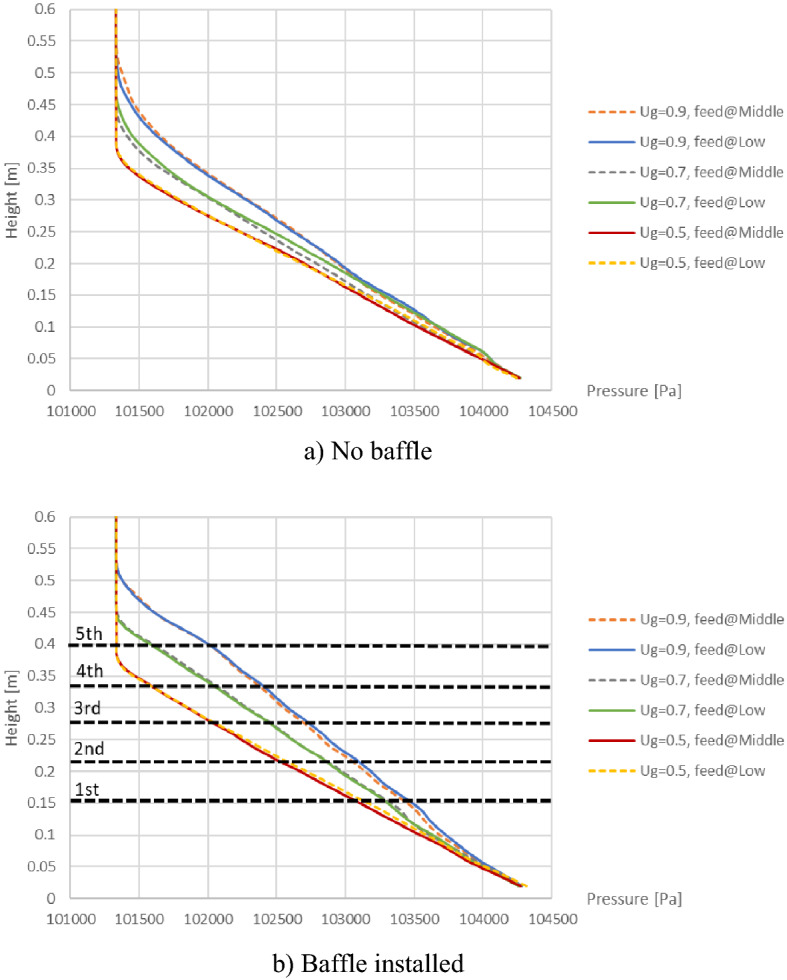
Comparison of time-and-area-averaged pressure when operating with different gas inlet velocities and different biomass feed positions.

In this part, the effect of baffle installation on the pressure drop across the bed and inside the bed was investigated. Figure 19 shows pressure profiles for various gas inlet velocities in cases before and after installing the baffles. Both with and without the baffles, all gas inlet velocities provide the same point of pressure near the bottom around 1.043 bar and the same line of pressure in the freeboard zone at 1.013 bar. Thus, these amounts of pressure drop across the bed were almost equal about 3.01–3.07 kPa. It accorded with the theory that the total pressure drop across the bed will be constant when the flow is in fully fluidized state^57,58^. However, the pressure lines in the bed zone would shift higher when operating with higher gas inlet velocity. In the same way, the pressure drop would be less at the same level in the bed zone. This is in accord with that the pressure drop in full fluidization depends on negative of bed voidage (${\upvarepsilon }_{\mathrm{L},\mathrm{ ff}}$) as shown in Eq. 26^58,59^, and the higher gas inlet velocity would provide bigger bubbles which increased voidage in bed.

27 $$\Delta {\text{P}}_{{{\text{L}},{\text{ff}}}} { = }\left( {1 - {\upvarepsilon }_{{{\text{L}},{\text{ff}}}} } \right)\left( {{\uprho }_{{\text{s}}} - {\uprho }_{{\text{g}}} } \right) \cdot {\text{g}} \cdot {\text{L}}$$

Furthermore, each line of biomass feeding at middle overlapped the line of the biomass feeding at bottom in every case. This indicated that position of biomass feed would not affect the pressure in bed at any height. It was also because the amount of biomass was very few compared to amount of bed.

**Figure 20:**
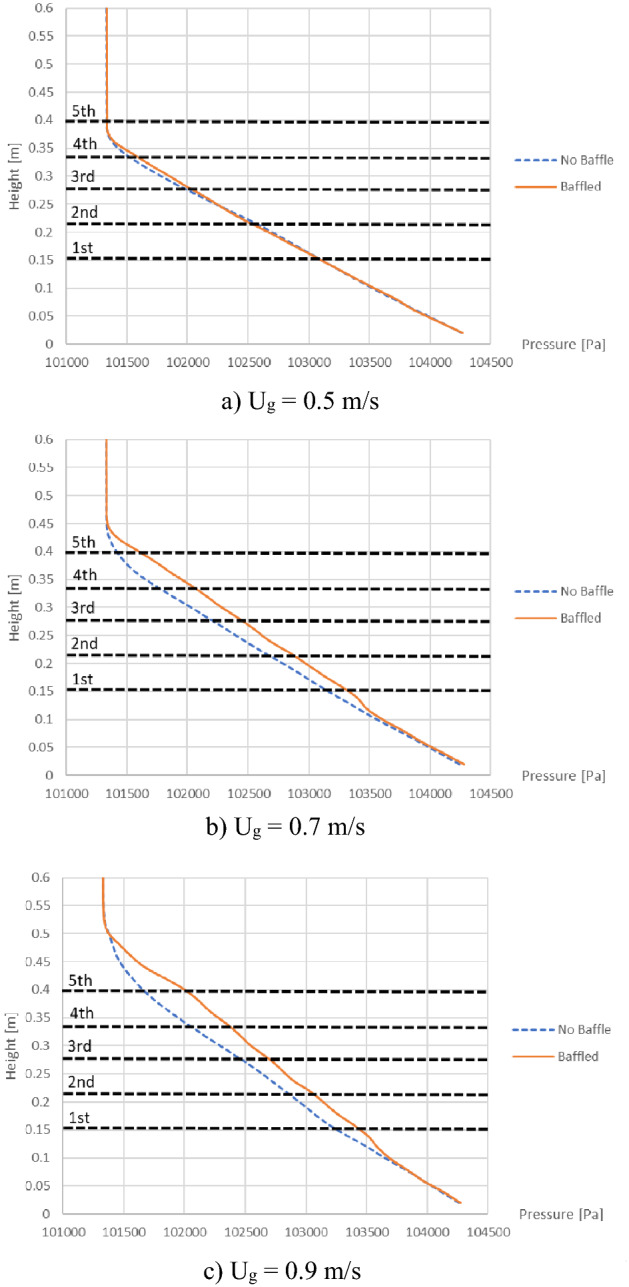
Time-and-area-averaged pressure before and after installing the baffles in case that biomass was fed at middle, when operating with various gas inlet velocities.

Then, for more obvious comparison between before and after installing the baffles, the cases of each gas inlet velocity were shown in Fig. 20. In case that gas inlet velocity was 0.5 m/s, the pressure lines were still overlapped to each other. But in cases that gas inlet velocities were 0.7 and 0.9 m/s, the pressure lines in cases of the baffles installed would shift slightly to right in the bed zone, in other words, the pressure drop at the same level would be slightly less. These might be due to a little increase of the voidage by the bubbles obstructed under the layer as previously discussed in Fig. 6.

In summary, installing the baffles had a little effect on the local pressure drop inside the bed, but it did not affect the total pressure drop across the bed. It meant that installing the baffles would not require additional energy for operation.

## Conclusion

In this study, the multilayers of louver baffles along the bed height were installed to reduce gas aggregation inside the bubbling bed gasifier of biomass. The Euler–Euler multiphase flow models with the kinetic theory of granular flow (KTGF) were applied for investigation. The results showed that the designed baffles could break and cut the bubbles. But it was found that the biomass should not be fed under the first baffle layer because biomass would be obstructed and lighten in the baffle zone. To improve this, the biomass feed channel should be at level in the middle of the baffle zone. In this case the biomass was still dense in the baffle zone.

The effect of the baffle installation on the biomass-bed mixing (Mixing index) and the gas-biomass contact (Contact index) was investigated. The baffles could provide good distribution of both the contact index and the mixing index in almost of bed zone, but both indices were significantly higher at a spot near the biomass feed channel.

Pressure in the bed zone was also investigated and found that the total pressure drop across the bed were equal with all gas inlet velocities and both with and without the baffles. That indicated no additional energy was required when the baffles were installed. However, when gas inlet velocity was higher, the pressure drop inside the bed would be slightly less if the baffles were installed.

Considering the effect of gas inlet velocity, the gas inlet velocity should be as less as possible to reduce gas aggregation. But the gas inlet velocity of 0.5 m/s was insufficient to blow the biomass fluently through the baffle layers and made the biomass bulked on top of bed. Thus, the performance of the gasifier would increase highest when the designed baffles were installed with biomass feeding at the middle of the baffle zone. The gas inlet velocity of 0.7 m/s could get the best performance of the provided system in this study.

## Data Availability

All data is included in this article. There is no supplemental data available in this article.

## List of symbols

CI: Contact index (–)
$${\text{C}}_{\text{D}}$$: Drag coefficient (–)
${\text{C}}_{\text{fr,ls}}$: Friction coefficient between solid phases (–)
${\text{d}}_{\text{s}}$: Particle diameter of solid phase (m)
${\text{e}}_{\text{ss}}\equiv{ \, {\text{e}}}_{\text{ls}}$: Restitution coefficient for solid–solid collisions (–)
${\text{G}}_{\text{s}}$: Inlet mass flux of solid, [kg/(m^2^ s)]
g, $$\overset{\lower0.5em\hbox{$\smash{\scriptscriptstyle\rightharpoonup}$}}{\text{g}}$$: Gravity force, (m/s^2^)
${\text{g}}_{\text{0,ls}}$: Radial distribution coefficient of mutual solid phases (–)
${\text{g}}_{\text{0,ss}}$: Radial distribution coefficient of single solid phase (–)
H: Static bed height (m)
${\text{H}}_{\text{g}}$: Specific enthalpy of gas phase (m^2^/s^2^, J/kg)
${\text{h}}_{\text{sg}}\equiv{ \, {\text{h}}}_{\text{gs}}$: Gas–solid interphase heat exchange coefficient (m^2^/s^2^, J/kg)
$$\bar{\text{I}}$$: Unit tensor (–)
$$\overset{\lower0.5em\hbox{$\smash{\scriptscriptstyle\rightharpoonup}$}} {\text{J}}_\text{k}^\text{q}$$: Mass flux of species k into phase q [kg/(m^2^ s)]
${\text{K}}_{\text{ls}}\equiv{ \, {\text{K}}}_{\text{sl}}$: Solid–solid interphase momentum exchange coefficient [kg/(m^3^ s)]
${\text{K}}_{\text{sg}}\equiv{ \, {\text{K}}}_{\text{gs}}$: Gas–solid interphase momentum exchange coefficient [kg/(m^3^ s)]
k_g_: Thermal conductivity of gas phase [W/(m K)]
$${\text{k}}_{{\Theta}_{\text{s}}}$$: Diffusion coefficient (m^2^/s)
L: Level in bed zone (m)
MI: Mixing index (–)
${\dot{\text{m}}}_{\text{pq}}$: Mass transfer from phase p to phase q [kg/(m^3^ s)]
Nu_s_: Nusselt number of solid phase (–)
Pr: Prandtl number of gas phase (–)
p: Static pressure (Pa)
p_q_: Static pressure of phase q (Pa)
p_s_: Solid pressure (Pa)
${\text{Q}}_{\text{sg}}$: Intensity of heat exchange between solid phase and gas phase (W/m^3^)
$$\overset{\lower0.5em\hbox{$\smash{\scriptscriptstyle\rightharpoonup}$}}{\text{q}}_{\text{g}}$$: Heat flux of gas phase (W/m^2^)
${\text{R}}_{\text{k}}^{\text{q}}$: Net rate of species k produced by homogeneous reactions inside phase q [kg/(m^3^ s)]
Re_s_: Particle Reynolds number of solid phase (–)
SD: Standard deviation (–)
${\text{S}}_{\text{h,g}}$: Heat source of gas phase (W/m^3^)
${\text{S}}_{\text{k}}^{\text{q}}$: Rate of creation of species k by addition from dispersed phase and other sources in phase q [kg/(m^3^ s)]
${\text{S}}_{\text{m,q}}$: Mass source of phase q [kg/(m^3^ s)]
Sc: Schmidt number (–)
U_g_: Gas inlet velocity (m/s)
U_pg_: Particle-gas relative velocity (m/s)
VF: Volume fraction (–)
$$\overset{\lower0.5em\hbox{$\smash{\scriptscriptstyle\rightharpoonup}$}}{\text{v}}_{\text{ls}}$$: Interphase velocity from phase l (solid or gas) to solid phase (m/s)
$$\overset{\lower0.5em\hbox{$\smash{\scriptscriptstyle\rightharpoonup}$}}{\text{v}}_{\text{q}}$$: Velocity of phase q (m/s)
$$\overset{\lower0.5em\hbox{$\smash{\scriptscriptstyle\rightharpoonup}$}}{\text{v}}_{\text{sg}}$$: Interphase velocity from solid phase to gas phase (m/s)
${\text{Y}}_{\text{k}}^{\text{q}}$: Mass fraction of species k in phase q (–)
${\upgamma}_{{\Theta}_{\text{s}}}$: Collisional dissipation of energy (W/m^3^)
$\Delta{\mathrm{P}}_{\mathrm{L},\mathrm{ ff}}$: Pressure drop at the level in bed zone when in full fluidized state (Pa)
${\upvarepsilon }_{\mathrm{L},\mathrm{ ff}}$: Bed voidage below the level when in full fluidized state (–)
ε_q_: Volume fraction of phase q (–)
${\upvarepsilon}_{\text{s,max}}$: Maximum packing of solid phase (–)
${\Theta}_{\text{s}}$: Granular temperature (m^2^/s^2^, J/kg)
${\lambda}_{\text{q}}$: Bulk viscosity of phase q (Pa s)
${\lambda}_{\text{s}}$: Solid bulk viscosity (Pa s)
${\upmu}_{\text{g}}$: Viscosity of gas [kg/(m s)]
${\upmu}_{\text{q}}$: Shear viscosity of phase q (Pa s)
${\upmu}_{\text{s}}$: Solid shear viscosity (Pa s)
${\upmu}_{\text{s,col}}$: Collisional viscosity of solid phase (Pa s)
${\upmu}_{\text{s,fr}}$: Friction viscosity of solid phase (Pa s)
${\upmu}_{\text{s,kin}}$: Kinetic viscosity of solid phase (Pa s)
ρ_g_: Density of gas (kg/m^3^)
ρ_q_: Physical density of phase q (kg/m^3^)
ρ_s_: Density of solid (kg/m^3^)
$\bar{\uptau}_{\text{q}}$: Stress tensor of phase q (Pa)
${\uptau}_{\text{s}}$: Particulate relaxation time in solid phase (s)
${\phi}_{\text{ls}}$: Kinetic energy exchange between phase l (solid or gas) and solid phase (W/m^3^)
